# Association of Aspirin Use with In-Hospital Mortality in Patients with Radiation-Associated Acute Pneumonitis: Insights from the National Inpatient Sample Database

**DOI:** 10.3390/healthcare14172827

**Published:** 2026-09-03

**Authors:** Akash Sharma, Shalini, Sowntappan Balasubramanian, Hamza Yousaf, Ankit Agrawal, Vrinda Vyas

**Affiliations:** 1 Department of Cardiovascular Medicine, University of Arkansas for Medical Sciences, Little Rock, AR 72205, USA; drankit.agrawal91@gmail.com; 2 Department of Medicine, University at Buffalo–Catholic Health System, Buffalo, NY 14214, USA; shalinigrover0001@gmail.com; 3 Department of Community Medicine, Seth G. S. Medical College and King Edward Memorial Hospital, Mumbai 400012, India; bsowntappan@gmail.com; 4 Department of Medicine, University of Missouri, Columbia, MO 65212, USA; hamzayousafnishter@gmail.com; 5 Cardiology, ECU Health, Tarboro, NC 27886, USA; vrinda.vyas@ecuhealth.org

**Keywords:** aspirin, chest radiation, mortality, heart failure

## Abstract

**Highlights:**

**What are the main findings?**

- Aspirin use is associated with reduced odds of in-hospital mortality among patients receiving chest radiation.
- This benefit was especially pronounced in patients with heart failure.

**What are the implications of the main findings?**

- Our study implies a potential protective mechanism with aspirin in patients with chest radiation.
- It is associated with decrease in mortality of radiation patients.

**Abstract:**

**Introduction:** This study investigates the relationship between long-term aspirin use and mortality in patients experiencing acute radiation-induced pulmonary complications. Methods: We conducted a secondary analysis from the National Inpatient Sample from 2016 to 2020. Our cohort comprised patients diagnosed with acute pneumonitis secondary to chest radiation. We adhered strictly to the STROBE checklist, extracting various patient characteristics for analysis. Categorical variables were compared using Pearson’s chi-square test, while continuous variables were assessed using Student’s two-sample t test. To assess the relationship, we employed multivariable-adjusted logistic regression, presenting results as odds ratios (ORs) with 95% confidence intervals (CIs). **Results:** The study included a total of 6064 hospitalizations: 5204 hospitalizations were in the not-taking-aspirin group (NAG), while 860 were in the aspirin group (AG). The mean age of the NAG cohort was 68.67 ± 12.31 years (mean ± SD), while for the AG it was 72.64 ± 8.87 (mean ± SD) years. Our multivariable analysis revealed that aspirin use is associated with 29% lower odds mortality (aOR = 0.71, 95% CI = 0.54–0.94, *p* = 0.02) than the NAG. Patients with heart failure (aOR = 0.34, 95% CI = 0.18–0.64, *p* < 0.001) showed the most significant mortality benefits. No mortality advantages of aspirin were observed in patients with coronary artery disease, percutaneous coronary intervention, or coronary artery bypass grafting. **Conclusions:** Aspirin use in patients with acute chest radiation is associated with decreased odds of mortality, particularly in those with a history of congestive heart failure.

## 1. Introduction

Radiation therapy serves as a primary treatment modality for various thoracic malignancies, including lung cancer, breast cancer, and Hodgkin’s lymphoma [1,2,3,4,5]. An estimated 10 to 30% of patients receiving radiation therapy develop radiation-associated heart disease in 5 years [6]. While effective in controlling cancer progression, exposure to chest radiation is associated with cardiovascular morbidity and mortality [1,2,3,4,5] and is an independent risk factor for mortality [7]. Lung cancer patients have 9% higher risk of death with radiation therapy as compared to no radiation therapy [7].

The adverse effects can cause acute and late cardiac injuries, including coronary artery disease (CAD), pericarditis, myocarditis, restrictive cardiomyopathy, valvular disease, conduction disturbances, and vasculopathy [1,2,3,4,5,8]. Pulmonary injuries include acute radiation pneumonitis and late pulmonary fibrosis [4]. These effects are driven by endothelial damage, inflammation, and oxidative stress [9,10,11]. Cardiovascular disease is now the most common non-malignancy cause of death in radiation-treated cancer survivors, most often occurring decades after treatment [3,10,11,12]. This underscores the pressing need for effective strategies aimed at preventing and mitigating cardiovascular events in patients receiving radiation therapy.

Notably, radiation therapy instigates the production of cytokines and reactive oxygen species (ROS), which contribute to sustained inflammation, tissue fibrosis, and the formation of atherosclerotic plaques [13,14]. Targeting these pathological processes with anti-inflammatory agents such as statins, aspirin, and colchicine presents a promising avenue for addressing radiation-associated cardiovascular disease (RACVD) and enhancing patient outcomes among those undergoing thoracic radiation [15].

Among these therapeutic options, aspirin stands out as a widely utilized antiplatelet agent with well-documented efficacy in the prevention and management of cardiovascular events. Its primary mode of action involves the irreversible inhibition of cyclooxygenase-1 (COX-1), leading to a reduction in thromboxane A2 levels. This action effectively prevents platelet aggregation, thereby mitigating thrombotic events. Furthermore, aspirin exhibits notable anti-inflammatory and antioxidant properties through the modulation of the prostaglandin pathway, suggesting potential applications in alleviating radiation-induced vascular and myocardial injuries.

Most research investigating the effects of aspirin in patients undergoing chest radiation has been limited to preclinical studies [16,17].

Despite the well-established role of aspirin in the management of cardiovascular disease, its role among patients receiving chest radiation therapy remains poorly defined. Existing evidence on aspirin use in the setting of radiation therapy is limited; even though there are studies, they are currently in their preclinical stage.

Therefore, further investigation is warranted to determine whether aspirin use is associated with any decrease in in-hospital mortality in this patient population. The aim of this study was to evaluate the association between aspirin use and in-hospital mortality among patients receiving chest radiation therapy.

## 2. Methods

### 2.1. Study Design and Variables

We adhered to the STROBE checklist while carrying out this study (Supplementary Table S1). This is an analytical retrospective cross-sectional study done by using the National Inpatient Sample (NIS) data, which was developed for the Healthcare Cost and Utilization Project. The NIS is the largest publicly available all-payer inpatient database, encompassing information from approximately 7 million hospital stays each year. For our research, we used NIS data from 2016 to 2020 [18]. NIS is a hospitalization-level database in which each record represents a single hospital discharge rather than a unique individual. Consequently, patients with multiple admissions may contribute more than one hospitalization to the dataset, and repeat hospitalizations cannot be identified.

As there is no specific code in the International Classification of Diseases (ICD) for chest radiation, we queried the NIS database for patients diagnosed with acute pulmonary complications (J70.0) resulting from chest radiation. Patients were identified using ICD-10-CM codes in the primary and secondary diagnosis fields. Patients using aspirin have been identified by the ICD code of long term/current use of aspirin (Z79.82). Additionally, we extracted other characteristics of the patient population, including age, sex, race, congestive heart failure, hyperlipidemia, coronary artery disease, and histories of percutaneous coronary intervention (PCI) and coronary artery bypass grafting (CABG), among others.

### 2.2. Statistical Analysis

The statistical analysis is performed using R statistical software (v.4.2, R Core Team 2024, Vienna, Austria). Categorical data are reported as counts with proportions and compared using Pearson’s chi-square test, while numerical data are presented as mean ± standard deviation (SD) and compared using Student’s two-sample t test justified by large sample size under the Central Limit Theorem. All statistical tests were 2-sided. To find the association between use of aspirin and in-hospital mortality, binomial multivariate logistic regression analysis was performed. Model fit and calibration were evaluated using the Hosmer–Lemeshow goodness-of-fit test, with predictions grouped into deciles of risk. Statistical significance was defined as a two-sided *p*-value < 0.05 [19,20]. Both unadjusted and adjusted logistic regression analyses were conducted, adjusting for factors such as age, sex, race, congestive heart failure, hyperlipidemia, coronary artery disease, history of PCI, CABG, income, diabetes mellitus, hypertension, obesity, smoking, atrial fibrillation, and end-stage renal disease.

In our research, we did not use NIS survey weights as our objective was to evaluate the association of aspirin with mortality in patients with chest radiation rather than estimating national population prevalence or total healthcare expenditures. However, to check the robustness of our results, we also conducted propensity score analysis. A 1:1 nearest-neighborhood propensity score matching was performed with a caliper of 0.2. Results were represented as odds ratios (ORs) or adjusted odds ratios (aORs) with 95% confidence intervals (CIs). *p*-value < 0.05 was considered significant. Subgroup analysis was also conducted based on patients’ comorbidities.

## 3. Results

In our study, 6064 patient hospitalizations are involved, of which 2926 (48.3%) were females. Out of total 6064 hospitalizations, 5204 hospitalizations were not taking aspirin and 860 were taking aspirin. There were 2580 (49.6%) females in the non-aspirin group (NAG) and 346 (40.3%) in the aspirin group (AG). The mean age of the NAG was 68.67 ± 12.31 (mean ± standard deviation (SD)), while for the AG it was 72.64 ± 8.87 (mean ± SD). In the NAG, 1328 (25.5%) patient hospitalizations involved congestive heart failure, while 253 (29.4%) did in the AG. A total of 23.2% of patient hospitalizations in the NAG involved coronary artery disease, while 49.2% did in the AG. Table 1 represents the different characteristics of the included population.

### Main Result

Logistic regression analysis was performed. The Hosmer and Lemeshow goodness-of-fit test *p*-value was 0.11, suggesting a good fit (no significant difference between observed and predicted value) of our model. Unadjusted analyses showed that the AG (OR = 0.63, 95% CI = 0.49–0.81, *p*-value < 0.01) has lower odds of in-hospital mortality in comparison to the NAG. Relation also holds true for the adjusted analysis (aOR = 0.71, 95% CI = 0.54–0.94, *p*-value = 0.02). To check the robustness of our results, we performed sensitivity analysis with propensity score matching. After matching the 860 hospitalizations in the AG with the NAG, logistic regression analysis on matched data showed patients with aspirin have lower odds (OR = 0.71, 95% CI = 0.51–0.98, *p*-value = 0.04) of in-hospital mortality as compared to the NAG. All the variables have optimal balance with standardized mean difference well below the standard threshold of <0.1.

**Figure 1:**
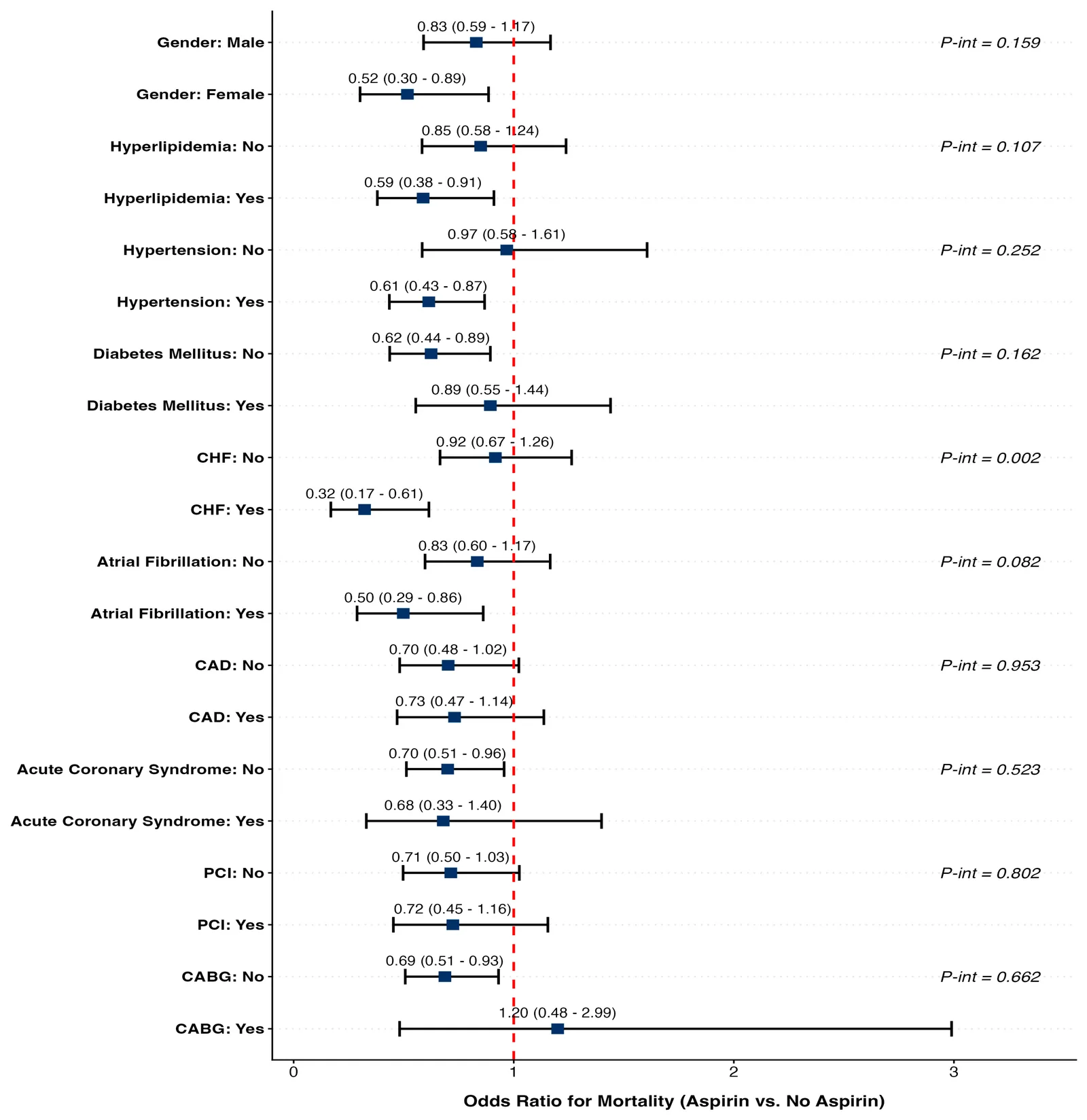
Interaction analysis of odds ratios for mortality (aspirin vs. no aspirin). Abbreviations: CHF, congestive heart failure; AF, atrial fibrillation; CAD, coronary artery disease; ACS, acute coronary syndrome; PCI, percutaneous coronary intervention; CABG, coronary artery bypass grafting.

Subgroup analysis was conducted based on the different comorbidities. Adjusted multivariable analysis shows that in the patient populations with congestive heart failure (aOR = 0.34, 95% CI = 0.18–0.64, *p*-value < 0.01) and atrial fibrillation (aOR = 0.49, 95% CI = 0.28–0.84, *p*-value = 0.01), the use of aspirin was associated with the highest decrease in odds of in-hospital mortality compared to the NAG. Even though the patients with atrial fibrillation have a mortality benefit, the interaction test (*p* = 0.08) proves that the treatment effect is not reliably different from patients without atrial fibrillation. However, in the patient populations with coronary artery disease (aOR = 0.76, 95% CI = 0.49–1.18, *p*-value = 0.22), PCI (aOR = 0.76, 95% CI = 0.48–1.20, *p*-value = 0.23), and CABG (aOR = 1.07, 95% CI = 0.45–2.54, *p*-value = 0.89), aspirin use was not associated with decrease in in-hospital mortality. Table 2 and Figure 1 represent the subgroup and interaction analyses.

## 4. Discussion

In this study, we aimed to evaluate the association between aspirin use and in-hospital mortality of patient hospitalizations with acute pneumonitis. We found that patients using aspirin had a higher mean age and a greater prevalence of comorbidities, including congestive heart failure (CHF), hyperlipidemia, coronary artery disease (CAD), history of percutaneous coronary intervention (PCI), coronary artery bypass grafting (CABG), end-stage renal disease (ESRD), and stroke. Despite the increased prevalence of these comorbidities, aspirin use was significantly associated with a reduction in in-hospital mortality. Additionally, we noted that female patients, those with CHF, hyperlipidemia, hypertension, and atrial fibrillation also experienced decreased in-hospital mortality with aspirin use. Because of the nature of the NIS data, we cannot determine the cause of death in our patient population.

Radiation therapy is known to contribute to coronary artery disease, valvular disease, cardiomyopathy, pericardial disease, and conduction abnormalities. Advances in radiation therapy techniques have reduced the risk of heart disease but have not eliminated it. Recent studies continue to show higher cardiovascular-related mortality among thoracic radiotherapy recipients than the general population [21,22], although recent landmark trials no longer recommend aspirin for most adults without established cardiovascular disease because the absolute reduction in myocardial infarction and ischemic stroke is generally balanced by an increased risk of major bleeding [23,24]. However, these trials have many fewer cancer patients among their participants. Given aspirin’s established role in both primary and secondary cardiovascular prevention via the inhibition of platelet aggregation and thrombus formation [25], it is plausible that a significant portion of the observed mortality benefit seen in our study is mediated through the attenuation of concurrent or secondary cardiovascular events.

Preclinical studies have proposed various hypotheses of radiation causing inflammatory damage to the heart. Radiation therapy alters various pathways, such as recruitment of cytokines and interleukins, leading to myocardial ischemia and fibrosis. It also activates reactive oxygen species and Rho signaling pathways, which result in accelerated atherosclerosis [15]. Current studies examining the effects of aspirin on radiation-associated cardiac injury are still in the preclinical stage. Some research suggests that aspirin may mitigate radiation-associated cardiovascular disease by reducing oxidative damage in irradiated rat models [26]; however, it has limitations, such as not preventing accelerated atherosclerosis in apolipoprotein E null rats [27]. Recent evidence indicates that aspirin may also affect the vascular endothelial growth factor (VEGF) pathway, which is impacted by radiation [28]. Furthermore, aspirin inhibits the expression of interleukin-1β, which is associated with a lower rate of cardiovascular events [29,30].

Further subgroup analyses suggested that the in-hospital mortality reduction associated with aspirin was most prominent in patients with CHF (aOR = 0.34). The beneficial effect of aspirin in patients with CHF might be attributed to the fact that these conditions are often secondary to myocardial ischemia. Research indicates that aspirin use in CHF may be associated with decreased mortality [31,32].

Interestingly, among patients with diabetes mellitus, male sex, CAD, PCI, and CABG, aspirin use was not associated with decreased in-hospital mortality. This suggests that the beneficial effects of aspirin in patients exposed to chest radiation are not solely due to the prevention of coronary artery disease; other pathways, such as the generation of reactive oxygen species, modulation of VEGF expression, and interleukin activity, may also play significant roles. In addition, these patients were on other therapies like statins and other platelet inhibitors that also have inflammatory properties and slow the atherosclerosis progression.

Several implications arise from this study. Most research regarding aspirin use in radiation-exposed patients is still in the preclinical stage. Our study provides a foundation for further clinical investigations, including randomized controlled trials. We found that even among patients taking aspirin who had a higher prevalence of various cardiac comorbidities, they demonstrated lower odds of in-hospital mortality after accounting for different variables. While primary prevention of heart disease secondary to radiation is challenging due to the extensive use of radiation therapy in various cancers, there is potential for more focused secondary prevention through aspirin use [33]. Secondary prevention may help manage associated cardiac diseases, and there are experimental studies suggesting that certain drugs, such as statins, ACE inhibitors, and antioxidants, can prevent radiation-induced heart disease (RIHD) [34,35,36].

This study has several limitations. We considered only hospitalized patients who developed radiation-associated injuries, who typically present with complications from chest radiation, potentially overlooking patients who received chest radiation but did not develop any complications. In our study, there are numerous subgroup evaluations, and interaction tests were uncorrected for multiplicity, meaning these secondary findings are exploratory and hypothesis-generating rather than definitive. Also, aspirin use likely identifies patients under cardiology care, receiving preventive therapies and under closer medical surveillance. Thus, aspirin may function as a surrogate marker for overall cardiovascular management. Furthermore, this is an observational study performed using the NIS and hence lacks granularity in data (comorbidities, cancer stage, and radiation therapy); therefore, a causal association between aspirin use and decreased in-hospital mortality cannot be definitively established. More robust studies, such as prospective cohort studies or randomized controlled trials, are needed to further illustrate this association.

## 5. Conclusions

Aspirin use was associated with lower odds of in-hospital mortality with radiation-associated acute pneumonitis, particularly among those with CHF. Prospective studies are needed to determine whether this association is causal.

## Figures and Tables

**Table 1:** Baseline characteristics of patients involved in our study.

	Non-Aspirin Group *n* (%) = 5204	Aspirin Group *n* (%) = 860	*p*-Value	SMD
Age (mean (SD))	68.67 (12.31)	72.64 (8.87)	<0.001	0.371
Died	607 (11.7)	66 (7.7)	0.001	0.135
Female	2580 (49.6)	346 (40.3)	<0.001	0.189
Race			0.002	0.173
1. White	3935 (77.6)	699 (82.9)		
2. Black	555 (10.9)	87 (10.3)		
3. Hispanic	284 (5.6)	31 (3.7)		
4. Asian or Pacific Islander	158 (3.1)	15 (1.8)		
5. Native American	26 (0.5)	2 (0.2)		
6. Other	115 (2.3)	9 (1.1)		
Income percentile (%)			0.963	0.02
1. 0–25th percentile	1230 (23.9)	200 (23.6)		
2. 26th to 50th percentile	1390 (27.1)	235 (27.8)		
3. 51st to 75th percentile	1327 (25.8)	220 (26.0)		
4. 76th to 100th percentile	1190 (23.2)	191 (22.6)		
Hyperlipidemia	1851 (35.6)	466 (54.2)	<0.001	0.381
Hypertension	3255 (62.5)	671 (78.0)	<0.001	0.344
Diabetes mellitus	1420 (27.3)	285 (33.1)	<0.001	0.128
Obesity	627 (12.0)	101 (11.7)	0.843	0.009
Smoking	2882 (55.4)	653 (75.9)	<0.001	0.443
Acute kidney injury	889 (17.1)	136 (15.8)	0.384	0.034
Acute renal failure	456 (8.8)	44 (5.1)	<0.001	0.144
Pulmonary hypertension	449 (8.6)	82 (9.5)	0.42	0.032
ESRD	59 (1.1)	13 (1.5)	0.437	0.033
Carotid artery disease	37 (0.7)	12 (1.4)	0.061	0.067
CHF	1328 (25.5)	253 (29.4)	0.018	0.087
Atrial fibrillation	1486 (28.6)	245 (28.5)	1	0.001
Cardiac arrythmia	846 (16.3)	164 (19.1)	0.045	0.074
CAD	1207 (23.2)	423 (49.2)	<0.001	0.562
ACS	323 (6.2)	66 (7.7)	0.121	0.058
Old MI	272 (5.2)	145 (16.9)	<0.001	0.378
PCI	1081 (20.8)	387 (45.0)	<0.001	0.534
CABG	215 (4.1)	110 (12.8)	<0.001	0.315
Permanent pacemaker	12 (0.2)	3 (0.3)	0.782	0.022
CVA	322 (6.2)	115 (13.4)	<0.001	0.244
Major stroke	106 (2.0)	22 (2.6)	0.391	0.035
Pericarditis	7 (0.1)	0 (0.0)	0.593	0.052
Myocarditis	2 (0.0)	0 (0.0)	1	0.028
Infective endocarditis	14 (0.3)	1 (0.1)	0.642	0.035

Abbreviations: SD, standard deviation; SMD, Standardized mean difference; ESRD, end-stage renal disease; CHF, congestive heart failure; CAD, coronary artery disease; ACS, acute coronary syndrome; MI, myocardial infarction; PCI, percutaneous coronary intervention; CABG, coronary artery bypass grafting; CVA, cerebrovascular accident.

**Table 2:** Subgroup and interaction analyses for mortality.

Subgroups	Strata	Unadjusted OR (95% CI)	Unadjusted *p*-Value	Adjusted aOR (95% CI) *	Adjusted *p*-Value *	Interaction *p*-Value
Gender (Male)	No	0.44 (0.26–0.73)	<0.01	0.52 (0.31–0.89)	0.02	0.159
	Yes	0.70 (0.51–0.96)	0.03	0.83 (0.59–1.16)	0.27	
Gender (Female)	No	0.70 (0.51–0.96)	0.03	0.83 (0.59–1.16)	0.27	0.159
	Yes	0.44 (0.26–0.74)	<0.01	0.52 (0.31–0.89)	0.02	
Hyperlipidemia	No	0.76 (0.53–1.08)	0.13	0.85 (0.58–1.24)	0.40	0.107
	Yes	0.53 (0.36–0.80)	<0.01	0.58 (0.38–0.90)	0.01	
Hypertension	No	0.87 (0.55–1.39)	0.57	0.97 (0.58–1.61)	0.91	0.252
	Yes	0.58 (0.42–0.80)	<0.01	0.62 (0.44–0.87)	0.01	
Diabetes Mellitus	No	0.56 (0.40–0.78)	<0.01	0.62 (0.44–0.89)	0.01	0.162
	Yes	0.80 (0.51–1.25)	0.33	0.89 (0.55–1.44)	0.63	
CHF	No	0.79 (0.58–1.06)	0.12	0.92 (0.67–1.26)	0.60	0.002
	Yes	0.33 (0.18–0.59)	<0.01	0.32 (0.17–0.61)	<0.01	
Atrial Fibrillation	No	0.73 (0.53–1.00)	0.05	0.83 (0.60–1.17)	0.30	0.082
	Yes	0.45 (0.28–0.75)	<0.01	0.50 (0.29–0.86)	0.01	
CAD	No	0.62 (0.43–0.89)	0.01	0.70 (0.48–1.02)	0.06	0.953
	Yes	0.68 (0.45–1.02)	0.06	0.73 (0.47–1.14)	0.17	
Acute Coronary Syndrome	No	0.60 (0.45–0.81)	<0.01	0.70 (0.51–0.96)	0.03	0.523
	Yes	0.68 (0.34–1.33)	0.25	0.68 (0.33–1.40)	0.29	
PCI	No	0.63 (0.44–0.89)	0.01	0.71 (0.50–1.03)	0.07	0.802
	Yes	0.68 (0.44–1.04)	0.08	0.72 (0.45–1.16)	0.18	
CABG	No	0.62 (0.47–0.83)	<0.01	0.69 (0.51–0.93)	0.02	0.662
	Yes	0.65 (0.29–1.44)	0.28	1.20 (0.48–2.99)	0.70	

* Adjusted for age, sex, race, congestive heart failure, hyperlipidemia, coronary artery disease, history of PCI, CABG, income, diabetes mellitus, hypertension, obesity, smoking, atrial fibrillation, and end-stage renal disease. Abbreviations: CHF, congestive heart failure; AF, atrial fibrillation; CAD, coronary artery disease; ACS, acute coronary syndrome; PCI, percutaneous coronary intervention; CABG, coronary artery bypass grafting.

## Data Availability

The study used data from the National Inpatient Sample, which are publicly available at https://hcup-us.ahrq.gov/databases.jsp (accessed on 23 July 2024).

## Supplementary Materials

The following supporting information can be downloaded at: https://www.mdpi.com/article/10.3390/healthcare14172827/s1, Table S1. STROBE Statement—checklist of items that should be included in reports of cross-sectional studies.

## Abbreviations

The following abbreviations are used in this manuscript: ESRD—end-stage renal disease; CHF—congestive heart failure; CAD—coronary artery disease; ACS—acute coronary syndrome; MI—myocardial infarction; PCI—percutaneous coronary intervention; CABG—coronary artery bypass graft; CVA—cerebro-vascular accident; OR—odds ratio; SD—standard deviation; RACVD—radiation-associated cardiovascular disease; COX—cyclooxygenase.
