# Supplementary material for: Association of Aspirin Use with In-Hospital Mortality in Patients with Radiation-Associated Acute Pneumonitis: Insights from the National Inpatient Sample Database
Source: Healthcare (Basel). 2026 Sep 3;14(17):2827. doi: 10.3390/healthcare14172827 (PMC13565229; doi:10.3390/healthcare14172827)
Supplement: Supplementary file 1 [file healthcare-14-02827-s001.zip › healthcare-4392825-supplementary.pdf]

Supplementary Table S1. STROBE Statement—checklist of items that should be included in reports of ***cross-sectional studies.***

	Item No	Recommendation	Page
**Title and abstract**	**1**	**(*a*) Indicate the study's design with a commonly used term in the title or the abstract.**	**1**
		**(*b*) Provide in the abstract an informative and balanced summary of what was done and what was found.**	**2**
**Introduction**			
**Background/rationale**	**2**	**Explain the scientific background and rationale for the investigation being reported.**	**4**
**Objectives**	**3**	**State specific objectives, including any prespecified hypotheses.**	**4**
**Methods**			
**Study design**	**4**	**Present key elements of study design early in the paper.**	**4**
**Setting**	**5**	**Describe the setting, locations, and relevant dates, including periods of recruitment, exposure, follow-up, and data collection.**	**4**
**Participants**	**6**	**(*a*) Give the eligibility criteria, and the sources and methods of selection of participants.**	**4**
**Variables**	**7**	**Clearly define all outcomes, exposures, predictors, potential confounders, and effect modifiers. Give diagnostic criteria, if applicable.**	**NA**
**Data sources/ measurement**	**8***	**For each variable of interest, give sources of data and details of methods of assessment (measurement). Describe comparability of assessment methods if there is more than one group.**	**4**
**Bias**	**9**	**Describe any efforts to address potential sources of bias.**	**4**
**Study size**	**10**	**Explain how the study size was arrived at.**	**NA**
**Quantitative variables**	**11**	**Explain how quantitative variables were handled in the analyses. If applicable, describe which groupings were chosen and why.**	**4**
**Statistical methods**	**12**	**(*a*) Describe all statistical methods, including those used to control for confounding.**	**4**
		**(*b*) Describe any methods used to examine subgroups and interactions.**	**4**
		**(*c*) Explain how missing data were addressed.**	**NA**
		**(*d*) If applicable, describe analytical methods taking account of sampling strategy.**	**NA**
		**(*e*) Describe any sensitivity analyses.**	**NA**
**Results**			

**Participants**	**13***	**(a) Report numbers of individuals at each stage of study—e.g., numbers potentially eligible, examined for eligibility, confirmed eligible, included in the study, completing follow-up, and analyzed.**	**NA**
		**(b) Give reasons for non-participation at each stage/**	**NA**
		**(c) Consider use of a flow diagram.**	**NA**
**Descriptive data**	**14***	**(a) Give characteristics of study participants (e.g., demographic, clinical, and social) and information on exposures and potential confounders.**	**5**
		**(b) Indicate the number of participants with missing data for each variable of interest.**	**5**
**Outcome data**	**15***	**Report numbers of outcome events or summary measures.**	**5**
**Main results**	**16**	**(*a*) Give unadjusted estimates and, if applicable, confounder-adjusted estimates and their precision (e.g., 95% confidence interval). Make clear which confounders were adjusted for and why they were included.**	**5 and table 2**
		**(*b*) Report category boundaries when continuous variables were categorized.**	**NA**
		**(*c*) If relevant, consider translating estimates of relative risk into absolute risk for a meaningful time period.**	**NA**
**Other analyses**	**17**	**Report other analyses done—e.g., analyses of subgroups and interactions, and sensitivity analyses.**	**5**
**Discussion**			
**Key results**	**18**	**Summarize key results with reference to study objectives.**	**6**
**Limitations**	**19**	**Discuss limitations of the study, taking into account sources of potential bias or imprecision. Discuss both the direction and magnitude of any potential bias.**	**7**
**Interpretation**	**20**	**Give a cautious overall interpretation of results considering objectives, limitations, multiplicity of analyses, results from similar studies, and other relevant evidence.**	**7**
**Generalizability**	**21**	**Discuss the generalizability (external validity) of the study results.**	**7**
**Other information**			
**Funding**	**22**	**Give the source of funding and the role of the funders for the present study and, if applicable, for the original study on which the present article is based.**	**8**
